# Cognitive enhancement of healthy older adults using hyperbaric oxygen: a randomized controlled trial

**DOI:** 10.18632/aging.103571

**Published:** 2020-06-26

**Authors:** Hadanny Amir, Daniel-Kotovsky Malka, Suzin Gil, Boussi-Gross Rahav, Catalogna Merav, Dagan Kobi, Hachmo Yafit, Abu Hamed Ramzia, Sasson Efrat, Fishlev Gregory, Lang Erez, Polak Nir, Doenyas Keren, Friedman Mony, Tal Sigal, Zemel Yonatan, Bechor Yair, Efrati Shai

**Affiliations:** 1 The Sagol Center for Hyperbaric Medicine and Research, Shamir (Assaf-Harofeh) Medical Center, Zerifin, Israel; 2 Sackler School of Medicine, Tel-Aviv University, Tel-Aviv, Israel; 3 The Mina and Everard Goodman Faculty of Life Sciences, Bar Ilan University, Ramat-Gan, Israel; 4 Radiology Department, Shamir Medical Center, Zerifin, Israel; 5 Research and Development Unit, Shamir Medical Center, Zerifin, Israel; 6 Sagol School of Neuroscience, Tel-Aviv University, Tel-Aviv, Israel

**Keywords:** aging, cognitive, hyperbaric oxygen, perfusion, cerebral blood flow

## Abstract

More than half of community-dwelling individuals sixty years and older express concern about declining cognitive abilities. The current study’s aim was to evaluate hyperbaric oxygen therapy (HBOT) effect on cognitive functions in healthy aging adults.

A randomized controlled clinical trial randomized 63 healthy adults (>64) either to HBOT(n=33) or control arms(n=30) for three months. Primary endpoint included the general cognitive function measured post intervention/control. Cerebral blood flow (CBF) was evaluated by perfusion magnetic resonance imaging.

There was a significant group-by-time interaction in global cognitive function post-HBOT compared to control (p=0.0017). The most striking improvements were in attention (net effect size=0.745) and information processing speed (net effect size=0.788).

Voxel-based analysis showed significant cerebral blood flow increases in the HBOT group compared to the control group in the right superior medial frontal gyrus (BA10), right and left supplementary motor area (BA6), right middle frontal gyrus (BA6), left middle frontal gyrus (BA9), left superior frontal gyrus (BA8) and the right superior parietal gyrus (BA7).

In this study, HBOT was shown to induce cognitive enhancements in healthy aging adults via mechanisms involving regional changes in CBF. The main improvements include attention, information processing speed and executive functions, which normally decline with aging.

## INTRODUCTION

More than half of community-dwelling individuals, sixty years and older, express concern about declining cognitive abilities [1]. Besides common pathological declines such as in Alzheimer’s dementia and mild cognitive impairments, normal cognitive aging is part of the normal aging process. Processing speed, conceptual reasoning, memory and problem-solving activities are the main domains which decline gradually over time [2]. Cerebrovascular dysfunction is an additional distinctive feature of aging that includes endothelial-dependent vasodilatation and regional decreases in cerebral blood flow (CBF) [3, 4]. Although not associated with a specific pathology, reduced regional CBF is associated with impaired cognitive functions [5, 6].

A growing body of research suggests several methods for cognitive enhancement and for improving the quality of life in both healthy and pathological states. Non pharmacological lifestyle interventions including exercise, healthy diets and cognitive training have shown positive effects if intensively performed [7, 8]. Unfortunately, so far, pharmacological interventions did not show significant improvements in cognitive performance in normal aging, and have significant risks for side effects [9].

Hyperbaric oxygen therapy (HBOT) utilizes 100% oxygen in an environmental pressure higher than one absolute atmospheres (ATA) to enhance the amount of oxygen dissolved in body’s tissues. Repeated intermittent hyperoxic exposures, has been shown to induce physiological effects which normally occur during hypoxia in a hyperoxic environment, including stem cells proliferation and generation of new blood vessels (angiogenesis) [10–13]. Angiogenesis is induced mainly in brain regions signaling ischemia or metabolic dysfunction [13–15]. In turn, neovascularization can enhance cerebral blood flow [14] and consequently improve the metabolic activity [13–15].

There is growing evidence from clinical studies that HBOT, utilized in a repeated daily sessions protocol, has neurotherapeutic effects which can improve cognitive functions in post-stroke, traumatic brain injury and anoxic brain damaged patients even years after the acute insult [15–19]. However, no study to date has examined HBOT’s neurocognitive effects in normal aging populations.

The aim of the current study was to evaluate whether HBOT affects cognitive function and brain perfusion in normal, non-pathological, aging adults.

## RESULTS

**Figure 1:**
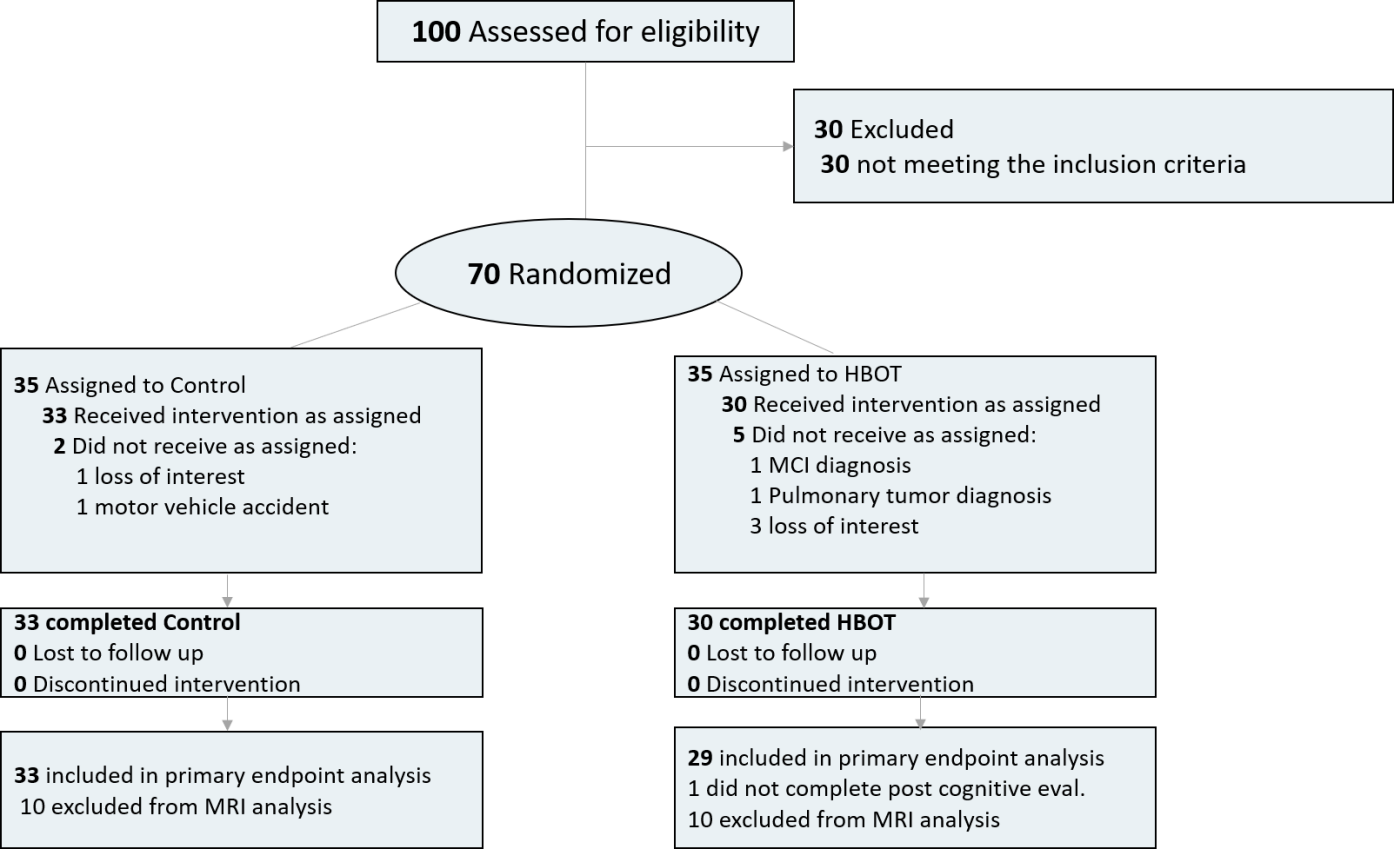
**Participants flowchart.**

Out of 100 individuals that were contacted for participation, 70 were eligible and signed an informed consent. Seven patients did not complete baseline assessments and were excluded All 63 patients who completed baseline evaluations completed interventions. One patient did not complete the cognitive assessment post-HBOT and excluded from analysis (Figure 1). The baseline characteristics and comparability of the cohort are provided in Table 1. The HBOT arm patients were slightly older (70.7±3.6 compared to 68.8±3.3) and apart from a higher rate of atrial fibrillation in the HBOT arm (4 patients 13.3% vs no patients 0%), there were no other significant differences between the two groups (Table 1).

**Table 1:** Baseline characteristics.

		**Total**	**Control**	**HBOT**	**P-value**
**N**		63 (100%)	33 (52.3%)	30 (47.7%)	
**Age (years)**		69.70±3.59	68.81±3.34	70.68±3.64	**0.041**
**Males**		39 (61.9%)	23 (69.7%)	16 (53.3%)	0.182
**Right dominance**		57 (90.5%)	2 (6.1%)	4 (13.3%)	0.326
**Life partner**		55 (87.3%)	29 (87.9%)	26 (86.7%)	0.885
**Education years**		15.23±2.81	15.03±2.87	15.45±2.78	0.564
**Working**		29 (46%)	16 (48.5%)	13 (43.3%)	0.682
**Family history**					
	Cognitive decline	23 (36.5%)	8 (24.2%)	15 (50%)	**0.034**
	Cancer	31 (49.2%)	16 (48.5%)	15 (50%)	
	Ischemic heart disease	12 (19%)	4 (12.1%)	8 (26.7%)	0.202
**Chronic medical conditions**					
	Atrial fibrillation	4 (6.3%)	0	4 (13.3%)	**0.046**
	Hypothyroidism	7 (11.1%)	3 (9.1%)	4 (13.3%)	0.593
	Obstructive sleep apnea	3 (4.8%)	0	3 (10%)	0.102
	Asthma	3 (4.8%)	2 (6.1%)	1 (3.3%)	1
	Benign prostatic hyperplasia	14 (22.2%)	7 (23.3%)	7 (21.2%)	0.842
	Gastroesophageal reflux disease	5 (7.9%)	2 (6.1%)	3 (10%)	0.662
	Osteoporosis	10 (15.9%)	5 (15.2%)	5 (16.7%)	0.869
	Rheumatic arthritis	4 (6.3%)	3 (9.1%)	1 (3.3%)	0.614
	Osteoarthritis	11 (17.5%)	4 (12.1%)	7 (23.3%)	0.325
	Diabetes mellitus	10 (15.9%)	7 (21.2%)	3 (10%)	0.308
	Hypertension	14 (22%)	7 (21.2%)	7 (23.3%)	0.84
	Dyslipidemia	30 (47.6%)	14 (42.4%)	16 (53.3%)	0.387
	Ischemic heart disease	6 (9.5%)	4 (12.1%)	2 (6.7%)	0.674
	History of smoking	24 (38.1%)	14 (42.4%)	10 (33.3%)	0.458
	Smoking pack years	22.38±13.33	21.21±10.75	24.0±16.79	0.625
	Quit smoking years	23.96±12.36	23.71±11.86	24.3±13.68	0.912
**Chronic medications**					
	Anti-aggregation	14 (22.2%)	6 (18.2%)	8 (26.7%)	0.418
	ACE-Inhibitors/ARB blockers	14 (22.2%)	8 (24.2%)	6 (20%)	0.686
	Beta blockers	11 (17.5%)	6 (18.2%)	5 (16.7%)	0.874
	Calcium blockers	6 (9.5%)	3 (9.1%)	3 (10%)	1
	Alpha blockers	13 (20.6%)	6 (18.2%)	7 (23.3%)	0.614
	Diuretics	3 (4.8%)	1 (3%)	2 (6.7%)	0.601
	Statins	19 (30.2%)	9 (27.3%)	10 (33.3%)	0.601
	Oral hypoglycemic	5 (7.9%)	4 (12.1%)	1 (3.3%)	0.357
	Bisphosphonates	4 (6.3%)	3 (9.1%)	1 (3.3%)	0.614
	Proton pump inhibitors	7 (11.1%)	4 (12.1%)	3 (10%)	1
	Hormones	4 (6.3%)	1 (3%)	3 (10%)	0.343
	PDE5-Inhibitors	11 (17.5%)	7 (21.2%)	4 (13.3%)	0.515
	Benzodiazepines	9 (14.3%)	6 (18.2%)	3 (10%)	0.479
	SSRI	8 (12.7%)	3 (9.1%)	5 (16.7%)	0.462

### Cognitive function

Results of the cognitive function evaluations are summarized in Tables 2, 3.

**Table 2:** Neurocognitive performance changes.

	***Control Group***			***HBOT Group***				
	***Baseline***	***Control***	***3 months P-value***	***Baseline***	***Post-HBOT***	***3 months P-value***	***Baseline Comparison P-value***	***Net Effect Size***
***Neurotrax***		**(N = 32)**			**(N = 29)**			
**Primary Endpoint**								
Global cognitive score	102.19±8.51	103.00±8.27	0.054	105.37±7.56	110.58±6.76	<0.000*	0.132	0.849
**Secondary Endpoints**								
Memory	105.20±7.54	105.53±7.10	0.757	104.23±10.53	108.46±7.01	0.004*	0.684	0.593
Verbal - Immediate	104.77±13.65	109.15±8.72	0.012	106.03±11.8	106.67±10.56	0.365	0.706	0.123
Verbal - Delayed	106.03±6.93	108.46±7.99	0.339	100.57±12.3	104.99±11.04	0.029*	0.037	0.293
Non-Verbal - Immediate	104.73±12.29	101.95±14.92	0.202	107.89±15.4	112.72±10.18	0.113	0.38	0.549
Non-Verbal - Delayed	101.78±14.59	100.27±13.43	0.513	103.82±12.34	109.46±10.18	0.035*	0.564	0.542
Executive Function	100.83±9.74	102.15±10.13	0.207	109.17±8.92	113.0±9.33	0.008*	<0.000*	0.381
Attention	99.96±7.81	101.10±6.96	0.247	102.89±9.66	108.90±6.51	<0.000*	0.196	0.745
Information Processing Speed	104.42±12.21	104.02±13.85	0.908	107.86±13.8	116.02±14.0	<0.000*	0.315	0.788
Motor Skills	100.29±11.43	99.90±10.98	0.746	104.63±11.2	107.79±9.03	0.075	0.145	0.445
***CANTAB***		**)N = 33)**			**(N = 29)**			
**Executive Function**								
ASTLCM(ms)	882.51±110.85	885.98±111.91	0.79	799.17±122.54	753.00±158.3	0.039	0.006	0.542
ASTLCMD(ms)	743.93±75.75	785.45±90.67	<0.000*	699.75±120.95	667.55±155.23	0.111	0.086	0.84
ASTLICM(ms)	961.48±121.99	963.37±128.53	0.903	884.51±139.61	823.35±161.90	0.009*	0.024	0.637
ASTLICMD(ms)	862.98±103.83	885.90±117.05	0.133	800.5±131.42	745.41±148.57	0.006*	0.041	0.861
***Pen and Paper***		**)N = 29)**			**(N = 28)**			
**Verbal memory**								
RAVLT total Z-score	0.31±0.85	0.32±0.88	0.969	0.09±1.08	0.61±0.94	0.062	0.395	0.603
**Executive function**								
Five points (percentile)	64.9±28.0	73.4±29.3	0.237	70.0±34.2	88.3±16.8	0.014	0.528	0.348
**Verbal fluency**								
F-A-S Z-Score (Semantic)	0.52±1.04	0.36±1.04	0.568	0.02±0.80	0.35±0.86	0.148	0.047	0.566

Baseline comparison p-value tests the null hypothesis of equal means of the two groups at the baseline using an unpaired t-test; 3 months comparison p-value tests the null hypothesis of equal means of each group pre-post intervention (HBOT/control respectively) using a paired t-test.

Bold - *P*<0.05, *Satisfied Bonferroni corrections.

Net effect size is the subtraction of Cohen’s D effect size of the control group from the HBOT group Cohen’s D effect size.

Neurotrax scores are normalized to age, gender and education years.

ASTLCM- The mean latency of response (from stimulus appearance to button press) on congruent trials.

ASTLCMD - The median latency of response (from stimulus appearance to button press) on congruent trials.

ASTLICM - The mean latency of response (from stimulus appearance to button press) on incongruent trials.

ASTLICMD - The median latency of response (from stimulus appearance to button press) on incongruent trials.

**Table 3:** Neurocognitive function repeated measures analysis.

	**Main Effect of Group**		**Main Effect of Time**		**Interaction Effect (Group_by_Time)**	
	***F***	***p-value***	***F***	***p-value***	***F***	***p-value***
***Neurotrax***						
**Primary Endpoint**						
Global Cognitive Score	7.171	0.009*	34.382	<0.000*	10.811	0.002*
**Secondary Endpoints**						
Memory	0.256	0.614	7.069	0.010*	5.186	**0.026**
Verbal – Immediate	0.195	0.66	4.602	**0.036**	0.220	0.64
Verbal - Delayed	4.61	**0.036**	5.732	0.02	1.216	0.274
Non-verbal - Immediate	5.511	0.002*	0.33	0.567	4.512	**0.037**
Non-verbal – Delayed	3.874	0.053	1.472	0.229	4.400	**0.04**
Executive Function	17.321	<0.000*	9.346	0.003*	2.213	0.142
Attention	8.688	0.004*	18.2	<0.000*	8.445	0.005*
Information Processing Speed	5.634	0.021*	8.082	0.006*	9.142	0.003*
Motor Skills	5.526	0.022*	1.781	0.187	2.964	0.09
*CANTAB*						
ASTLCM	12.716	0.001*	3.408	0.07	4.458	**0.039**
ASTLCMD	8.980	0.004*	0.033	0.857	10.702	0.002*
ASTLICM	10.563	0.002*	5.488	**0.023**	6.146	**0.016**
ASTLICMD	11.183	0.001*	2.262	0.138	11.254	0.001*
*Pen and Paper*						
RAVLT Total Z-Score	0.059	0.809	6.876	**0.011**	5.439	**0.023**
Five Points (Percentile)	2.400	0.127	16.641	**<0.000**	1.778	0.188
F-A-S Z-Score (Semantic)	1.449	0.234	1.233	0.271	4.646	**0.035**

Using a 2X2 repeated measures ANOVA model, the cognitive scores were compared between the 2 groups.

The first two columns present the between group effect. The 3^rd^ and 4^th^ columns report the time repeated effect (within group). The 5^th^ and 6^th^ columns report the group-by-time interaction;

Bold – P<0.05, *-Satisfied Bonferroni corrections;

Neurotrax scores are normalized to age, gender and education years.

### Primary endpoint

**Figure 2:**
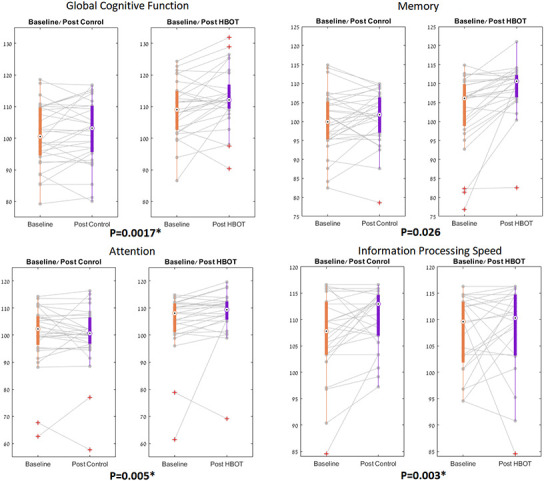
**NeuroTrax parameters significant changes.** The NeuroTrax significant domains, shown in boxplots, with each line representing a patient flow from baseline to post intervention. The central mark indicates the median, and the bottom and top edges of the box indicate the 25^th^ and 75^th^ percentiles, respectively. Red symbols indicate outliers.

Both groups had similar global cognitive scores at baseline which was higher than the average score normalized to age and education level (>100). There was a significant group by time interaction in the primary endpoint of global cognitive function post HBOT compared to the control group (F=10.811, p=0.0017 with a net effect size of 0.849 (Tables 2, 3 and Figure 2).

### Secondary endpoints

Both groups had similar cognitive scores in all domains measured by Neurotrax at baseline, The most striking improvements were in attention (net effect size=0.745, F=8.445, p=0.005) and information processing speed (net effect size=0.788, F=9.142, p=0.003) (Figure 2). Both overall memory domain score, the immediate and the delayed nonverbal memory scores were all improved post-HBOT compared to the control group (Tables 2, 3 and Figure 2). However, they were statistically insignificant following multi-comparisons corrections.

**Figure 3:**
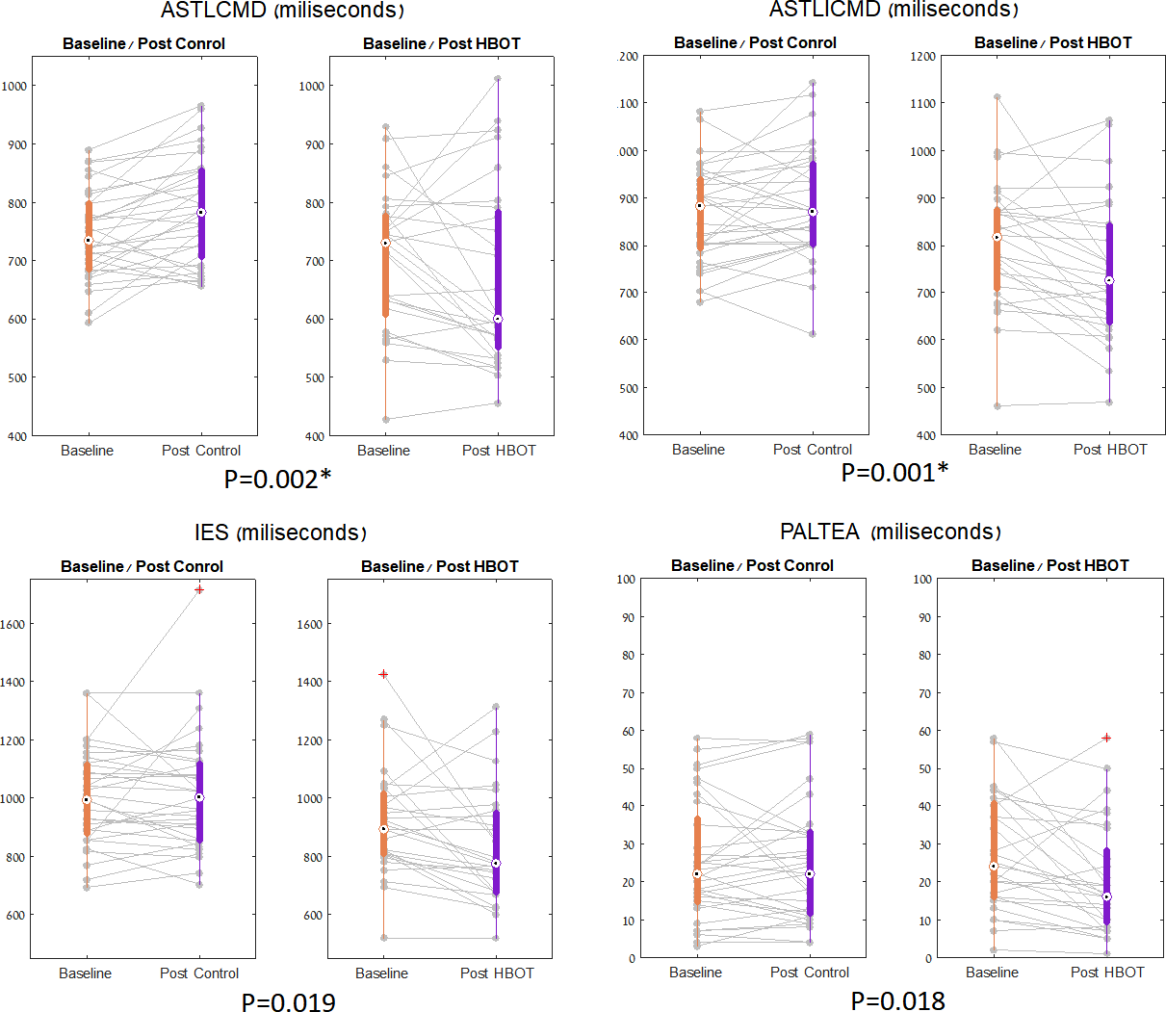
**CANTAB parameters significant changes.** The set shifting parameters, shown in boxplots, with each line representing a patient flow from baseline to post intervention. The central mark indicates the median, and the bottom and top edges of the box indicate the 25^th^ and 75^th^ percentiles, respectively. Red symbols indicate outlie.

In the CANTAB battery, at baseline, the HBOT group had shorter response times. However, they were insignificant following multiple comparison corrections (Table 2). The HBOT group showed significant improvement in the set shifting task performance (a subset of executive function) (Tables 2, 3 and Figure 3). There was a significant reduction in median response time in both congruent (net effect size=-0.861, F=10.702, p=0.002) and incongruent trials (net effect size=-0.840, F=11.254, p=0.001) following HBOT (Tables 2, 3 and Figure 3). There was an increased performance in the visual memory task post-HBOT, which was statistically insignificant following multiple comparisons corrections. The results of covariate-adjusted (age and education) analyses were similar.

In the traditional paper and pencil tasks, at baseline, both groups showed similar cognitive functions in all tasks. Both verbal fluency (FAS semantic, net effect size=0.566, F=4.646, p=0.03) and verbal memory (RAVLT total, net effect size=0.603, F=5.439, p=0.02) improved in the HBOT group, compared to the control group. However, they were statically insignificant following multiple comparisons corrections (Table 2, 3). (See all cognitive results in Supplementary Table 1, Supplementary Table 2).

### Brain perfusion

Ten subjects were excluded due to excessive head motion (>1mm or 1°) and ten subjects were excluded due to low AIF peak or wide AIF. A total of 20 subjects from the control group and 19 subjects from the HBOT group were included in the analysis.

There was an insignificant increase in whole brain CBF (p=0.054) and whole grey matter CBF (p=0.057) and no significant group by time interaction in whole brain, grey matter and white matter CBF (p>0.05) (Supplementary Table 3).

**Figure 4:**
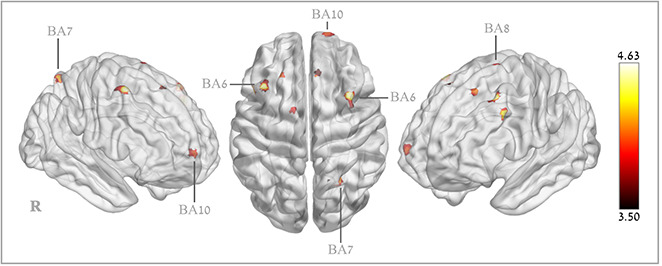
**Brain regions with significant post hyperbaric oxygen therapy changes in cerebral blood flow.**

Voxel-based analysis revealed significant CBF increases in the HBOT group compared to the control group in the following regions: right superior medial frontal gyrus (BA 10), right and left supplementary motor area (BA 6), right middle frontal gyrus (BA 6), left middle frontal gyrus (BA9), left superior frontal gyrus (BA8) and the right superior parietal gyrus (BA 7) (Table 4 and Figure 4).

**Table 4:** Brain regions with significant perfusion increases.

**Anatomical location**	**BA**	**Coordinates**			**t-value**
		**X**	**Y**	**Z**	
Right superior medial frontal gyrus	6	10	30	64	4.63
Right supplementary motor area	6	8	24	70	3.72
Left supplementary motor area	6	-14	0	78	3.92
Right middle frontal gyrus	6	38	12	60	4.56
Right middle frontal gyrus	6	38	4	82	3.8
Left middle frontal gyrus	9	-38	22	52	4.52
Left middle frontal gyrus	8	-26	32	56	4.13
Right superior frontal gyrus	10	18	68	8	4.04
Right superior parietal gyrus	7	-48	18	38	4.41
Right superior parietal gyrus	7	24	-62	60	4.21

The tables report each brain region which was found significant in a time-by-group repeated measures ANOVA comparing the two groups. The results are showing in specific Montreal Neurological Institute (MNI) coordinates; X, sagittal, Y, coronal, Z, axial, refers to Montreal Neurological Institute. BA, Brodmann area.

All coordinates emerged at a threshold of *P* < 0.05, corrected; minimum cluster size: 25 (200 mm^3^).

There were moderate correlations between cognitive score changes and these regional perfusion changes. The Neurotrax memory score change correlated to the left middle frontal gyrus CBF change (BA8), (r=0.379, p=0.023) and the attention score change correlated to the right middle frontal gyrus (BA6) (r=0.339, p=0.043). CANTAB set switching parameter changes correlated with the right superior frontal gyrus and supplementary motor area (BA6) (r=0.38-0.45, p<0.05, Supplementary Table 4). The RAVLT total score correlated with both the right superior medial frontal gyrus (r=0.393, p=0.016), right supplementary motor area (r=0.394, p=0.016) and the right superior parietal gyrus (r=0.380, p=0.002) (Table 4). The FAS semantic score change correlated with the right medial frontal gyrus (r=0.353, p=0.03) (Supplementary Table 4).

### Quality of life

At baseline, there were no significant differences in all quality of life domains as calculated from the SF-36 questionnaire. All but the energy domain, had high normal values (>75) in both groups. There was a significant increase in the HBOT group’s energy levels, compared to none in the control group (Supplementary Tables 5, 6).

### Safety

Four participants (13.3%) experienced mild middle ear barotrauma (TEED 1-2) in the HBOT group compared to none (0%) in the control group. All events were treated conservatively and all participants completed their protocol. Fifteen participants (62% of those without intraocular lens implantation) had visual acuity changes in the HBOT group, compared to ten (37% of those without intraocular lens implantation). No changes were found in participants who had intraocular lens surgery prior to the study. In the HBOT group, nine patients (31.0%) had far sight acuity deterioration while six patients (20.6%) had improvement in their far sight acuity. Six patients (20.6%) had improved near sight acuity and three (10.3%) had near sight acuity deterioration. Four participants (16.7%) in the HBOT group, compared to nine (33.3%) in the control group had cataract level acceleration.

## DISCUSSION

We found HBOT can induce significant enhancements in cognitive performance in healthy elderly. The main improved domains include attention, information processing speed and executive function (set shifting) in addition to global cognitive functions. Moreover, the HBOT group had a significantly enhanced brain perfusion in the superior and middle frontal gyri, supplementary motor area and superior parietal lobule.

Attention and information processing speed were significantly increased following HBOT compared to no change in the control group. In executive functions, the particular subset of set shifting improved significantly following HBOT compared to actual deterioration in the control group. These domains are known to gradually decline within the process of normal aging and play a crucial role in the daily functioning of the elderly [2]. Our results were in participants that had neither previous brain pathologies nor pathological cognitive declines (such as dementia or mild cognitive impairment) and had normal baseline cognitive scores. The memory domain, and mainly the non-verbal subdomain, improved in the HBOT group compared to no change in the control group. However, these changes did not reach corrected significance. We cannot rule out the possibility that in larger sample sizes, the memory domain effect would remain significant even after multi-comparisons corrections. Interestingly, the most significant cognitive changes were found in the computerized cognitive batteries and not in the traditional psychometric pen and paper tools. This may be due to the lack of proper alternative variations for the traditional tasks and low test-retest validity [20].

The HBOT net effect size of global cognitive score enhancement was 0.849 following 12 weeks HBOT sessions, substantially more than in other enhancement modalities. Colcombe et al. in a meta-analysis found that aerobic training in older adults improves mostly executive function (task switching) followed by improvements in spatial and speed, and an overall improvement, with an effect size of d=0.47 [21]. Recently, a randomized controlled trial showed only the executive function moderately improving following aerobic training (d=0.36) with no significant changes in memory, language and verbal fluency domains (d=0.12-0.15) [8]. In contrast, our study shows that HBOT has a significant large net effect size of 0.84-0.86 in the global cognitive score, task switching, as well as a significant net effect size in verbal fluency, attention, and information processing speed.

Previous studies have demonstrated a single oxygen exposure can enhance the cognitive function such as verbal function, visuospatial function through increased brain activation [22–26]. In a recent study, multitasking was significantly enhanced during hyperbaric oxygen exposure [27]. Yu et al. recently showed that five hyperbaric sessions enhance healthy young adults’ spatial memory, correlated with increased functional connectivity in the hippocampus, inferior frontal gyrus and lingual gyrus [28]. However, these changes were evaluated the day after the last hyperbaric session. In comparison, our intervention included 60 sessions within three months and to exclude transient effects of oxygen, all measurements were performed at least one week after the last hyperbaric session.

The current study HBOT protocol utilized the effects induced by repeated intermittent hyperoxic exposures, the so called hyperoxic hypoxic paradox [10]. These intermittent hyperoxic exposures induce many of the physiological responses that occur during hypoxia [10]. HBOT induces the release of the transcription factors called hypoxic induced factor (HIF) and increases their stability and activity [11]. In turn, HIF-1α and HIF-2α modulate the release of the angiogenic factor vascular endothelial growth factor (VEGF) [29, 30]. VEGF is considered the master regulator of angiogenesis, and induces migration of progenitor endothelial cells from the bone marrow into the circulatory system, recruitment of endothelial cells from existing blood vessels and the differentiation into new formed blood vessels [31]. Importantly, the migration of these circulating angiogenic cells targets sites of ischemia where they promote vascular remodeling and stimulate angiogenesis [32]. As seen in ischemic brain injuries, neovascularization increases regional cerebral blood flow [14, 31, 33, 34]. We suggest that repeated oxygen level fluctuations may improve regional CBF and cognitive functions in healthy elderly.

Our protocol included 60 sessions of 100% oxygen at 2 ATA including 3 air breaks during each session in order to utilize the hyperoxic hypoxic paradox and minimize the risk for oxygen toxicity. However, the dose response curve related to the applied pressure, time and number of HBOT exposures and its relation to HIF expression and its related regenerative effects are still not fully understood and further studies are needed to find the optimal HBOT protocols.

The significant improvement in CBF induced by HBOT in the current study population was in certain cortical regions. This finding is in agreement with the work by Martin et al. which demonstrated age-related functional decline is related to reduced perfusion in specific cortical locations rather and not the global CBF, but rather in the cortical regions that are the most sensitive for the age-related functional decline [4]. Recently, another study confirmed the selective age-related reductions in cortical perfusion [35]. Following HBOT, the increase in the CBF was in specific regions which participate in the following cognitive roles:

Superior medial frontal gyrus (SFG) (BA 10) - is thought to contribute to higher cognitive functions and has mostly been associated multitasking, attention, social cognition and episodic memory [36, 37].

Middle frontal gyrus (MFG) - has been proposed as a site of convergence of the dorsal and ventral attention networks. The MFG serves as a gateway to interrupt ongoing endogenous attentional processes in the dorsal attention network and reorient attention to an exogenous stimulus [38, 39].

Premotor cortex (BA6) - The premotor area and supplementary motor area (SMA) functions include motor sequencing and planning movements. It has been shown that area 6 participates in memory, attention and executive function as well as updating verbal function and updating spatial information [40].

Superior frontal gyrus (BA8) – traditionally, this area has been regarded as the frontal eye field. However, functional studies have shown significant participation of this area in executive function (including reasoning and planning), working memory and attention [41–43].

Superior parietal lobule (BA7) – also referred as the somatosensory association cortex (together with BA5), is believed to play a role in visuo-motor coordination and attention. In addition, it seems to participate in semantic categorization tasks and temporal context recognition [44].

### Study limitations

The current study has several limitations and strengths to consider. First, the limited sample size has to be taken into account, possibly causing decreased sensitivity and false negative changes. However, the presence of significant changes following strict statistical analyses in a small group is indicative for the relatively high potency of the intervention. Second, the control group was a non-intervention rather than a sham-intervention. Although the outcome assessors were blinded, the participants were unblinded. Third, the duration of the effect is yet to be determined in long-term follow-ups. Nevertheless, several strengths should be stressed. The isolated HBOT effect was measured as both groups were monitored for any lifestyle changes (such as nutrition and exercise), medications or any other intervention that may have acted as possible confounders. Patients did not perform any cognitive training tasks during the trial, thereby excluding training effects. Both computerized cognitive batteries had alternate forms with test-retest validity as well as the brain perfusion sequence and analysis. Moreover, the improvements in the cognitive domains correlated with the significant changes in perfusion MRIs.

In summary, the study indicates that HBOT can induce cognitive enhancement in healthy aging populations. The main improvements include attention, information processing speed and executive functions, which are known to decline with normal aging. In correlation with the cognitive improvements, HBOT induced a significant brain perfusion increase in specific brain regions with high cognitive roles.

## MATERIALS AND METHODS

### Subjects

Seventy adults without pathological cognitive declines, aged 64 and older, who lived independently in good functional and cognitive status were enrolled. The study was performed between 2016-2020 in the Shamir (Assaf-Harofeh) Medical Center, Israel. Included patients did not have cardiac or cerebrovascular ischemia histories for the last year prior to inclusion. Exclusion criteria included: previous treatment with HBOT for any reason during the last three months, any history of malignancy during the last year, any pathological cognitive decline, severe chronic renal failure (GFR <30), uncontrolled diabetes mellitus (HbA1C>8, fasting glucose>200), immunosuppressants, MRI contraindications, active smoking and pulmonary diseases.

Included patients who were diagnosed with pathological cognitive decline based on their cognitive tests were excluded. Pathological cognitive decline was diagnosed by a certified neuropsychologist.

### Study design

The study protocol was approved by Institutional Review Board of Shamir medical center, Israel. The study was performed as a randomized, prospective controlled clinical trial. After signing an informed consent, the subjects were assigned either to HBOT or control (no intervention) arms. Assessors were blinded to the participants’ intervention assignment. Measurement points were evaluated at baseline and 1-2 weeks after the HBOT or control period.

### Interventions

The HBOT protocol was administrated in a multiplace Starmed-2700 chamber (HAUX, Germany). The protocol comprised of 60 daily sessions, 5 sessions per week within a three month period. Each session included breathing 100% oxygen by mask at 2ATA for 90 minutes with 5-minute air breaks every 20 minutes. Compression/ decompression rates were 1 meter/minute. The control arm received no active intervention as a no-contact group. During the trial, neither lifestyle and diet changes, nor medications adjustments were allowed for either group.

### Cognitive measures

Cognitive functions were assessed using two computerized batteries and one traditional paper-based battery, given by a certified neuropsychologist.

**NeuroTrax** computerized cognitive testing battery (NeuroTrax Corporation, Bellaire, TX). The NeuroTrax system and a detailed description of the tests included were detailed in previous publications [45–47] and are also available on the NeuroTrax website (http://www.neurotrax.com/).

In brief, the NeuroTrax tests evaluate multiple aspects of brain cognitive functions including: memory, executive function (EF), attention, information processing speed (IPS), motor skills (MS) visuospatial skills (VS) and verbal function (VF). Cognitive domain scores were normalized for age, gender and education-specific levels.

The participants completed validated alternate test forms of the NeuroTrax test battery at baseline and post-HBOT, to allow for iterative administrations with minimal learning effects. Test-retest reliability of the tests were found to be high in both normal and injured populations, without significant learning effects except in the VF and VS domains that were not evaluated in the current study [48, 49].

**CANTAB** computerized cognitive tests (Cambridge cognition, England) [50]. CANTAB is a semiautomated test battery which can be administered on a handheld tablet. The battery included: attention switching tasks (AST) for executive function and set shifting testing, pair associates learning (PAL) for evaluating visual memory and new learning, reaction time (RTI), rapid visual information processing (RVP) for assessing sustained attention, spatial span (SSP) for assessing visuospatial working memory and spatial working memory (SWM) [51, 52]. To combine accuracy and reaction time, the inverse efficiency score (IES) was calculated by the following formula:

$$IES=\frac{response\;time}{1-percentage\;of\;errors}$$

Notably, the patients were given different test versions of the CANTAB test battery at baseline and after the control/HBOT period, to allow repeated administrations with minimal learning effects. The current version of CANTAB has no population norms for either parameter.

**Traditional paper and pencil based** neuropsychological tests included: the Rey-Osterrieth complex figure test (ROCFT), a popular measure of visuoconstructive skills and visual memory [53]; the Rey auditory verbal learning test (RAVLT), a neuropsychological assessment designed to evaluate verbal memory in patients [54]; the digit symbol substitution test (DSST) offers high sensitivity to detect overall cognitive impairment rather than a specific domain [55]; a digit-span (DS) task, used to measure working memory’s number storage capacity [56]; the five points test (5PT) is a structured and standardized test that assesses figural fluency functions which are associated with executive functioning [57]; the trails making test (TMT), a widely used test that assesses organized visual search, planning, attention, set shifting, cognitive flexibility, and divided attention, all capacities thought to be executive in nature [58]; the FAS test, which measures phonemic word fluency, which is a type of verbal fluency [59]; the bells test, a cancellation test, which permits qualitative and quantitative evaluation of visual neglect [60]. Scores were normalized to age, gender and education as suggested in the manuals and presented as Z-scores. In case of diagnosed pathological cognitive decline using both the cognitive scores and MMSE, the patients were excluded from the study.

### Brain MRIs

MRI scans were performed on a MAGNETOM Skyra 3T scanner, configured with20-channel receiver head coils (Siemens Healthcare, Erlangen, Germany). The MRI protocol included dynamic susceptibility contrast (DSC), and post-contrast high-resolution MPRAGE 3D T1-weighted images.

MRI sequences parameters:

***DSC:*** Fifty T2*-weighted gradient-echo echo planar imaging (EPI) volumes were acquired, two repetitions before a bolus injection of gadolinium-DTPA (Gd-DTPA, 0.2 ml/kg, administered at 5 ml/sec), 48 repetitions after injection of Gd-DTPA. Sequence parameters: TR: 2,300 ms, TE: 40ms, flip angle: 30°, voxel size :1.8 x1.8, matrix: 128x128, number of slices: 25, and slice thickness = 3.9 mm.

***MPRAGE*** **3D:** was acquired in sagittal orientation with 0.9 mm isotropic resolution. Sequence parameters: TR: 2,000 ms, TE: 2.41 ms, flip angle: 8°, TI: 928 ms, FOV: 245 x 245, and 192 contiguous slices.

### DSC-MRI analysis

The preprocessing of the perfusion MRI data was preformed using the SPM software (version 12, UCL, London, UK) and included motion correction, and co-registration with MPRAGE T1 images. Individual gray matter (GM) and white matter (WM) segmentation of T1 anatomy was also performed to extract mean perfusion values. Whole-brain quantitative perfusion analysis was performed as described in previous studies [61, 62]. Detailed description is found in the supplementary material (SI-1). Briefly, MR signal intensity was converted to Gd concentrations, AIF was determined automatically, fitted to the gamma variate function and deconvolved on a voxel-by-voxel basis to calculate the CBF, CBV, and MTT maps. Following normalization to MNI space, WM and GM masking and smoothing using a 6 mm full-width at half-maximum Gaussian kernel, statistical analysis was performed on the normalized CBF maps, using the voxel-based method.

### Quality of life measures

The RAND health status survey, short form-36 (SF-36) was used to assess quality of life. SF-36 is a self-report measure that evaluates physical functioning, bodily pain, role limitations due to physical health problems, role limitations due to personal or emotional health, general mental health, social functioning, energy/fatigue, and general health perception [63–65]. Each scale generates a score from 0 to 100, with a high score indicating better health and less body pain.

### Safety

Participants were monitored for adverse events including: barotraumas (either ear or sinuses), oxygen toxicity (pulmonary and central nervous system). Participants were examined by a certified blinded ophthalmologist before and after the control/HBOT term to monitor visual acuity and cataracts.

### Statistical analysis

Continuous data were expressed as means ± standard-deviation. The normal distribution for all variables was tested using the Kolmogorov-Smirnov test. unpaired and paired t-tests were performed to compare variables between and within the two groups. Net effect sizes were evaluated using Cohen’s d method.

Continuous parameters correlations were performed using Pearson and Spearman’s as appropriate.

Categorical data is expressed in numbers and percentages and compared by chi-square tests. Univariate analyses were performed using Chi-Square/Fisher’s exact test to identify significant variables (P<0.05).

To evaluate HBOT’s effects on cognitive scores, a within-subject repeated measures ANOVA model was used to test the main interaction effect between time and group. The false discovery rate (FDR) method was used for multiple comparisons correction. In addition, covariate-adjusted effects were examined, with covariates of age, sex and years of education.

To evaluate HBOT’s effects on CBF, statistical analysis was performed on the normalized CBF maps, using the voxel-based method implemented in SPM12 (Wellcome Trust Centre for Neuroimaging, London, England). A within-subject repeated measure ANOVA model was used to test the main interaction effect between time and group, using the SPM factorial model. The statistical significance level was set to a voxel-wise *P*-value of 0.05 corrected for multiple comparisons using the sequential Hochberg correction [66], with a minimum cluster size of 25 (200 mm3) contiguous significant voxels. CBF values in each cluster were extracted and averaged.

Statistical significance threshold was set to 0.05. Data were statistically analyzed using MatLAB 2018b (Mathworks, Natick, MA).

### Sample size

Based on previous studies on cognitive improvements following HBOT, assuming a five-point improvement in the global cognitive score in NeuroTrax following HBOT, compared to two points in the control group, with a four point standard deviation of the change, with a power of 80% and an alpha of 5%, 29 participants would be required in each arm. Adding a 15% dropout rate would require 70 patients in total.

## Supplementary Material

Supplementary Methods

Supplementary Table 1

Supplementary Tables
