# Supplementary material for: Cognitive enhancement of healthy older adults using hyperbaric oxygen: a randomized controlled trial
Source: Aging (Albany NY). 2020 Jun 26;12(13):13740–61. doi: 10.18632/aging.103571 (PMC7377835; doi:10.18632/aging.103571)
Supplement: Supplementary Methods [file aging-12-103571-s002..pdf]

SUPPLEMENTARY METHODS

SI-1: MRI analysis

DSC analysis steps:

1. Conversion of signal intensity to concentration of Gd-DTPA with respect to time:

$$C_m(t) = -K * \ln\left(\frac{S(t)}{S_0}\right)$$

where Cm(t) is the measured concentration of Gd-DTPA with respect to time, K is a proportionality constant that is inversely proportional to the TE and depends on the MR scanner, S(t) is the MRI signal intensity with respect to time, and so is the baseline MRI signal before the presence of Gd-DTPA and after a steady-state magnetization has been achieved [3].

2. Arterial input function: the AIF was measured automatically, using the following algorithm:

   a. The volume with maximum Cm(t) intensity was identified (10th-13th volume). Only voxels with maximum intensity in this volume were identified as AIF candidates.

   b. Only voxels with maximum intensity higher than the 96th percentile and lower than the 99.9^{th} percentile were included.

   c. Only voxels with a shape of sharp increase and sharp decrease were included.

   d. The AIF voxel candidates were fitted to the gamma variate function using the following equation [3]. Goodness of fit was evaluated and only voxels with $R^2 > 0.96$ were included.

$$AIF_{fit}(t)\, or\, C_{fit}(t) = -K(x-\Delta)^{\alpha} * e^{-\frac{x-\Delta}{B}} * F_{step}(x-\Delta)$$

   e. The final AIF was an average of the Cm(t) signal in the voxels passing the above criteria.

   f. Normalization of AIF: To allow a uniform time of injection in all subjects and DSC scans, the Cm(t) was shifted in case of early/late injection to allow a uniform AIF peak at the 10th volume.

3. Gamma fitting of AIF and Cm: The AIF and Cm(t) were fitted to the gamma variate function using the gamma fit equation (see above) [3].

where AIFfit(t) and Cfit(t) are the fitted AIF(t) and Cm(t) curves, respectively, K is a constant, x is the image number, Δ is the delay between image 0 and the arrival of the bolus (a positive number), α and B are gamma variate parameters, and Fstep is a step function defined by:

$$F_{step} = \begin{cases} 1 \; for \; (x-\Delta) \geq 0 \\ 0 \; for (x-\Delta) < 0 \end{cases}$$

4. SVD deconvolution: The fitted AIF was used to calculate C(t) (the tissue response to an instantaneous arterial bolus) using SVD deconvolutions was done by Ostergaard et al. (1996). In short, the values for the AIF and Cm(t) curves can be written in vector notation as C = AIF−1 • Cm, where C represents the matrix of the deconvolved C(t) curve. This equation can be solved using the SVD technique, whereby the matrix AIF is decomposed into three matrices AIF = U • W • VT. The inverse of AIF can be calculated as AIF^{-1} = V • [diag(1/wj)] • UT, where [diag(1/wj)] represents the reciprocals of the diagonal elements of W. When calculating AIF^{-1}, problems arise when W contains singular values (i.e., wj = 0 or is close to 0) and will cause the curve C(t) to oscillate. Therefore, we used a cutoff threshold of 10% [1].

5. Calculation of CBV was performed based on the fitted Cm(t) and AIF:

$$CBV = \frac{k}{\rho} * \frac{\int C_m(t)\,dt}{\int AIF(t)\,dt}$$

where κ = (1 − HCTLV)/(1 − HCTSV) corrects for the fact that the hematocrit in large vessels (HCTLV was set to 0.45) is larger than the hematocrit of small vessels (HCTSV was set to 0.25) (1) and ρ is the density of brain tissue (1.04 g/ml) [3].

6. Calculation of CBF was performed using the following equation:

$$\frac{CBV}{CBF} = \frac{\int C(t)\,dt}{C_{max}}$$

where C(t) is the concentration of Gd-DTPA in a tissue region and Cmax is the maximum of this curve [3].

7. MTT was calculated [2]:

$$MTT = \frac{CBV}{CBF}$$

8. Normalization of the CBF: Since the amount of injection was not uniform between scans, the CBF was normalized using a factor of 1.9 divided by the AIF peak value.

Supplementary References

1. Ostergaard L, Sorensen AG, Kwong KK, Weisskoff RM, Gyldensted C, Rosen BR. High resolution measurement of cerebral blood flow using intravascular tracer bolus passages. Part II: Experimental comparison and preliminary results. Magn Reson Med. 1996; 36:726–36. https://doi.org/10.1002/mrm.1910360511 PMID:8916023

2. Ostergaard L, Weisskoff RM, Chesler DA, Gyldensted C, Rosen BR. High resolution measurement of cerebral blood flow using intravascular tracer bolus passages. Part I: Mathematical approach and statistical analysis. Magn Reson Med. 1996; 36:715–25. https://doi.org/10.1002/mrm.1910360510 PMID:8916022

3. Smith AM, Grandin CB, Duprez T, Mataigne F, Cosnard G. Whole brain quantitative CBF, CBV, and MTT measurements using MRI bolus tracking: implementation and application to data acquired from hyperacute stroke patients. J Magn Reson Imaging. 2000; 12:400–10. https://doi.org/10.1002/1522-2586(200009)12:3<400::aid-jmri5>3.0.co;2-c PMID:10992307
