# Supplementary material for: Cognitive enhancement of healthy older adults using hyperbaric oxygen: a randomized controlled trial
Source: Aging (Albany NY). 2020 Jun 26;12(13):13740–61. doi: 10.18632/aging.103571 (PMC7377835; doi:10.18632/aging.103571)
Supplement: Supplementary Table 1 [file aging-12-103571-s001..docx]

**Supplementary Table 1. Neurocognitive performance changes.**

|  | ***Control Group*** | | | ***HBOT Group*** | | |  |  |
| --- | --- | --- | --- | --- | --- | --- | --- | --- |
|  | ***Baseline*** | ***Control*** | ***3 months***  ***P-value*** | ***Baseline*** | ***Post-HBOT*** | ***3 months***  ***P-value*** | ***Baseline Comparison P-value*** | ***Net Effect Size*** |
| ***Neurotrax*** | | **)N = 32)** |  |  | **(N = 29)** |  |  |  |
| **Primary Endpoint** | | |  |  |  |  |  |  |
| Global cognitive score | 102.19±8.51 | 103.00±8.27 | 0.054 | 105.37±7.56 | 110.58±6.76 | <0.000* | 0.132 | 0.849 |
| Secondary Endpoints | | |  |  |  |  |  |  |
| Memory | 105.20±7.54 | 105.53±7.10 | 0.757 | 104.23±10.53 | 108.46±7.01 | 0.004* | 0.684 | 0.593 |
| Verbal - Immediate | 104.77±13.65 | 109.15±8.72 | 0.012 | 106.03±11.8 | 106.67±10.56 | 0.365 | 0.706 | 0.123 |
| Verbal - Delayed | 106.03±6.93 | 108.46±7.99 | 0.339 | 100.57±12.3 | 104.99±11.04 | 0.029* | 0.037 | 0.293 |
| Non-Verbal - Immediate | 104.73±12.29 | 101.95±14.92 | 0.202 | 107.89±15.4 | 112.72±10.18 | 0.113 | 0.38 | 0.549 |
| Non-Verbal - Delayed | 101.78±14.59 | 100.27±13.43 | 0.513 | 103.82±12.34 | 109.46±10.18 | 0.035* | 0.564 | 0.542 |
| Executive Function | 100.83±9.74 | 102.15±10.13 | 0.207 | 109.17±8.92 | 113.0±9.33 | 0.008* | <0.000* | 0.381 |
| Attention | 99.96±7.81 | 101.10±6.96 | 0.247 | 102.89±9.66 | 108.90±6.51 | <0.000* | 0.196 | 0.745 |
| Information Processing Speed | 104.42±12.21 | 104.02±13.85 | 0.908 | 107.86±13.8 | 116.02±14.0 | <0.000* | 0.315 | 0.788 |
| Motor Skills | 100.29±11.43 | 99.90±10.98 | 0.746 | 104.63±11.2 | 107.79±9.03 | 0.075 | 0.145 | 0.445 |
| CANTAB | | )N = 33) |  |  | (N = 29) |  |  |  |
| Executive Function | | |  |  |  |  |  |  |
| ASTLCM(ms) | 882.51±110.85 | 885.98±111.91 | 0.79 | 799.17±122.54 | 753.00±158.3 | 0.039 | 0.006 | 0.542 |
| ASTLCMD(ms) | 743.93±75.75 | 785.45±90.67 | <0.000* | 699.75±120.95 | 667.55±155.23 | 0.111 | 0.086 | 0.84 |
| ASTLICM(ms) | 961.48±121.99 | 963.37±128.53 | 0.903 | 884.51±139.61 | 823.35±161.90 | 0.009* | 0.024 | 0.637 |
| ASTLICMD(ms) | 862.98±103.83 | 885.90±117.05 | 0.133 | 800.5±131.42 | 745.41±148.57 | 0.006* | 0.041 | 0.861 |
| ASTLDM(ms) | 965.05±107.42 | 989±112.24 | 0.157 | 881.24±133.17 | 834.17±166.80 | 0.039 | 0.008 | 0.684 |
| ASTLM(ms) | 921.71±113.21 | 924.25±115.81 | 0.847 | 841.5±127.67 | 788.10±158.61 | 0.017 | 0.011 | 0.617 |
| IES(ms) | 998.9±165.8 | 1013.6±202.4 | 0.587 | 916.7±185.5 | 830.8±192.4 | 0.016 | 0.07 | 0.619 |
| **Visual Memory and Learning** | | |  |  |  |  |  |  |
| PALTEA | 25.15±15.64 | 25.18±16.3 | 0.987 | 27.31±14.18 | 20.48±14.51 | 0.011* | 0.58 | 0.576 |
| PALTEA8 | 13.09±9.35 | 15.42±8.68 | 0.248 | 16.86±9.45 | 13.10±9.00 | 0.026 | 0.222 | 0.657 |
| **Reaction Time** | | |  |  |  |  |  |  |
| RTIFMRT(ms) | 434.7±66.4 | 444.4±63.1 | 0.112 | 396.5±61.0 | 390.0±56.1 | 0.549 | 0.022 | 0.348 |
| RTIFMDRT(ms) | 427.4±65.3 | 433.4±59.8 | 0.305 | 383.8±62.9 | 379.7±55.2 | 0.694 | 0.009 | 0.22 |
| RVPMDL(ms) | 471.6±84.3 | 472.3±81.7 | 0.948 | 433.1±93.6 | 418.4±85.5 | 0.226 | 0.094 | 0.242 |
| **Visuospatial working memory** |  |  |  |  |  |  |  |  |
| SSPFSL | 5.3±0.99 | 5.68±1.02 | 0.069 | 5.3±0.96 | 5.7±0.82 | 0.161 | 0.927 | -0.04 |
| SSPRSL | 5.21±1.05 | 5.28±1.17 | 0.488 | 5.13±1.15 | 5.26±1.22 | 0.791 | 0.792 | -0.04 |
| SWMBE | 15.06±6.9 | 13.25±6.4 | 0.075 | 16.03±6.8 | 15.1±7.6 | 0.749 | 0.593 | 0.281 |
| ***Pen and Paper*** | | **)N = 29)** |  |  | **(N = 28)** |  |  |  |
| **Visual memory** |  |  |  |  |  |  |  |  |
| ROCFT Z-score (immediate) | 0.9±0.72 | 0.89±0.63 | 0.949 | 1.06±0.66 | 1.11±0.61 | 0.791 | 0.372 | 0.001 |
| ROCFT Z-score (delayed) | 0.83±0.79 | 1.19±0.75 | 0.067 | 1.09±0.87 | 1.42±0.74 | 0.136 | 0.22 | 0.024 |
| Digit symbol substitution test Z-score | 0.18±0.65 | 0.4±0.84 | 0.277 | 0.25±0.83 | 0.85±0.84 | 0.026 | 0.765 | 0.547 |
| **Working memory** |  |  |  |  |  |  |  |  |
| Digit span Z-score | 0.02±0.81 | -0.04±0.72 | 0.736 | -0.11±0.55 | 0.09±0.77 | 0.319 | 0.493 | 0.489 |
| **Verbal memory** |  |  |  |  |  |  |  |  |
| RAVLT total Z-score | 0.31±0.85 | 0.32±0.88 | 0.969 | 0.09±1.08 | 0.61±0.94 | 0.062 | 0.395 | 0.603 |
| **Executive function** |  |  |  |  |  |  |  |  |
| Five points (percentile) | 64.9±28.0 | 73.4±29.3 | 0.237 | 70.0±34.2 | 88.3±16.8 | 0.014 | 0.528 | 0.348 |
| Trails B (Z-score) | -0.27±1.04 | 0.14±0.52 | 0.048 | -0.1±1.58 | 0.14±0.66 | 0.449 | 0.609 | 0.139 |
| Bells (errors) | 2.2±2.7 | 1.7±1.6 | 0.381 | 3.5±4.8 | 2.9±2.9 | 0.573 | 0.187 | 0.079 |
| **Verbal fluency** |  |  |  |  |  |  |  |  |
| F-A-S Z-Score (Semantic) | 0.52±1.04 | 0.36±1.04 | 0.568 | 0.02±0.80 | 0.35±0.86 | 0.148 | 0.047 | 0.566 |

Baseline comparison p-value tests the null hypothesis of equal means of the two groups at the baseline using an unpaired t-test; 3 months comparison p-value tests the null hypothesis of equal means of each group pre-post intervention (HBOT/control respectively) using a paired t-test. Bold - *P*<0.05, *Satisfied Bonferroni corrections

Net effect size is the subtraction of Cohen's D effect size of the control group from the HBOT group Cohen's D effect size.

Neurotrax scores are normalized to age, gender and education years.

ASTLCM- The mean latency of response (from stimulus appearance to button press) on congruent trials.

ASTLCMD - The median latency of response (from stimulus appearance to button press) on congruent trials.

ASTLICM - The mean latency of response (from stimulus appearance to button press) on incongruent trials

ASTLICMD - The median latency of response (from stimulus appearance to button press) on incongruent trials

ASTLDM - The mean latency of response (from stimulus appearance to button press), calculated for trials where the instruction was to respond to the direction of the stimulus

ASTLM - The mean latency of response (from stimulus appearance to button press), calculated across all correct, assessed trials

PALTEA - The number of times the subject chose the incorrect box for a stimulus on assessment problems (PALTE), plus an adjustment for the estimated number of errors they would have made on any problems, attempts and recalls they did not reach

PALTEA8 - The number of times the subject chose the incorrect box for a stimulus on assessment problems, where the number of shapes was equal to 8 (PAL total errors (8 shapes)), plus an adjustment for the estimated number of errors they would have made on any problems, attempts and recalls (with shapes equal to 8) they did not reach

RTIFMRT - The mean duration between the onset of the stimulus and the release of the button. Calculated for correct, assessed trials where the stimulus could appear in any one of five locations

RTIFMDRT - The median duration between the onset of the stimulus and the release of the button. Calculated for correct, assessed trials where the stimulus could appear in any one of five locations

RVPMDL - The median response latency during assessment sequence blocks where the subject responded correctly

SSPFSL - The longest sequence successfully recalled by the subject. Forwards variant only

SSPRSL -The longest sequence successfully recalled by the subject. Reverse variant only

SWMBE - Between errors are defined as times the subject revisits a box in which a token has previously been found. This is calculated for trials of four, six and eight tokens
