# Supplementary material for: Cognitive enhancement of healthy older adults using hyperbaric oxygen: a randomized controlled trial
Source: Aging (Albany NY). 2020 Jun 26;12(13):13740–61. doi: 10.18632/aging.103571 (PMC7377835; doi:10.18632/aging.103571)
Supplement: Supplementary Tables [file aging-12-103571-s003..pdf]

## SUPPLEMENTARY TABLES

**Supplementary Table 2. Neurocognitive function repeated measures analysis.**

| | Main Effect of Group | | Main Effect of Time | | Interaction Effect (Group_by_Time) | |
|---|---|---|---|---|---|---|
| | *F* | *p-value* | *F* | *p-value* | *F* | *p-value* |
| ***Neurotrax*** | | | | | | |
| **Primary Endpoint** | | | | | | |
| Global Cognitive Score | 7.171 | **0.009*** | 34.382 | **<0.000*** | 10.811 | **0.002*** |
| **Secondary Endpoints** | | | | | | |
| Memory | 0.256 | 0.614 | 7.069 | **0.010*** | 5.186 | **0.026** |
| Verbal – Immediate | 0.195 | 0.66 | 4.602 | **0.036** | 0.220 | 0.64 |
| Verbal - Delayed | 4.61 | **0.036** | 5.732 | **0.02** | 1.216 | 0.274 |
| Non-verbal - Immediate | 5.511 | **0.002*** | 0.33 | 0.567 | 4.512 | **0.037** |
| Non-verbal – Delayed | 3.874 | 0.053 | 1.472 | 0.229 | 4.400 | **0.04** |
| Executive Function | 17.321 | **<0.000*** | 9.346 | **0.003*** | 2.213 | 0.142 |
| Attention | 8.688 | **0.004*** | 18.2 | **<0.000*** | 8.445 | **0.005*** |
| Information Processing Speed | 5.634 | **0.021*** | 8.082 | **0.006*** | 9.142 | **0.003*** |
| Motor Skills | 5.526 | **0.022*** | 1.781 | 0.187 | 2.964 | 0.09 |
| ***CANTAB*** | | | | | | |
| ASTLCM | 12.716 | **0.001*** | 3.408 | 0.07 | 4.458 | **0.039** |
| ASTLCMD | 8.980 | **0.004*** | 0.033 | 0.857 | 10.702 | **0.002*** |
| ASTLICM | 10.563 | **0.002*** | 5.488 | **0.023** | 6.146 | **0.016** |
| ASTLICMD | 11.183 | **0.001*** | 2.262 | 0.138 | 11.254 | **0.001*** |
| ASTLDM | 14.911 | **<0.000*** | 0.98 | 0.326 | 7.104 | **0.01** |
| ASTLM | 11.98 | **0.001*** | 4.855 | **0.031** | 5.770 | **0.019** |
| IES | 9.217 | **0.003*** | 3.023 | 0.087 | 5.822 | **0.019** |
| PALTEA | 0.124 | 0.726 | 3.022 | 0.08 | 5.822 | **0.019** |
| PALTEA8 | 0.023 | 0.88 | 1.208 | 0.276 | 6.677 | **0.012** |
| RTIFMRT | 10.019 | **0.002*** | 0.077 | 0.782 | 1.878 | 0.176 |
| RTIFMDRT | 11.321 | **0.001*** | 0.029 | 0.864 | 0.782 | 0.38 |
| RVPMDL | 5.125 | **0.027** | 0.744 | 0.392 | 0.907 | 0.345 |
| SSPFSL | 0.038 | 0.847 | 5.441 | **0.023** | 0.030 | 0.863 |
| SSPRSL | <0.0001 | 0.989 | 0.388 | 0.536 | 0.025 | 0.874 |
| SWMBE | 1.156 | 0.287 | 2.293 | 0.136 | 1.106 | 0.298 |
| ***Pen and Paper*** | | | | | | |
| ROCFT Z-Score (immediate) | 2.326 | 0.133 | 0.22 | 0.641 | 0 | 0.995 |
| ROCFT Z-Score (delayed) | 1.496 | 0.226 | 14.503 | **<0.000** | 0.009 | 0.925 |
| Digit symbol substitution test(Z) | 1.451 | 0.234 | 17.317 | **<0.000** | 3.598 | 0.064 |
| Digit Span (Z-score) | 0.000 | 0.986 | 0.537 | 0.467 | 2.872 | 0.097 |
| RAVLT total (Z-score) | 0.059 | 0.809 | 6.876 | **0.011** | 5.439 | **0.023** |
| Five Points (percentile) | 2.400 | 0.127 | 16.641 | **<0.000** | 1.778 | 0.188 |
| Trials B (Z-score) | 0.191 | 0.664 | 3.694 | 0.06 | 0.282 | 0.597 |
| F-A-S Z-score (Semantic) | 1.449 | 0.234 | 1.233 | 0.271 | 4.646 | **0.035** |

Using a 2X2 repeated measures ANOVA model, the cognitive scores were compared between the 2 groups. The first two columns present the between group effect. The 3rd and 4th columns report the time repeated effect (within group). The 5th and 6th columns report the group-by-time interaction;
Bold – P<0.05, *-Satisfied Bonferroni corrections. Neurotrax scores are normalized to age, gender and education years.

**Supplementary Table 3. Global cerebral blood flow changes.**

| | *Control Group (N=19)* | | | *HBOT Group (N=20)* | | | | |
|---|---|---|---|---|---|---|---|---|
| | ***Baseline*** | ***Control*** | ***3 months P-value*** | ***Baseline*** | ***Post-HBOT*** | ***3 months P-value*** | ***Baseline Comparison P-value*** | ***Group by time P-value*** |
| Whole Brain | 41.34±6.22 | 41.55±6.78 | 0.88 | 47.13±7.69 | 50.22±7.26 | 0.054 | 0.014 | 0.180 |
| Grey Matter | 47.79±8.84 | 47.93±9.89 | 0.94 | 58.20±9.43 | 61.92±8.22 | 0.057 | 0.001 | 0.170 |
| White Matter | 28.40±7.35 | 28.57±7.17 | 0.88 | 29.12±6.37 | 30.67±4.93 | 0.198 | 0.747 | 0.472 |

**Supplementary Table 4. Significant correlations between cognitive changes and perfusion changes.**

| **Area** | **BA** | **Battery** | **Test** | **Parameter** | **R** | **P-Value** |
|---|---|---|---|---|---|---|
| Right superior medial frontal gyrus | 6 | CANTAB | Set shifting | ASTLCM | -0.343 | 0.03 |
| Right superior medial frontal gyrus | 6 | CANTAB | Set shifting | ASTLCD | -0.384 | 0.01 |
| Right superior medial frontal gyrus | 6 | CANTAB | Set shifting | ASTLMD | -0.455 | 0.004 |
| Right superior medial frontal gyrus | 6 | CANTAB | Set shifting | ASTLICM | -0.41 | 0.01 |
| Right superior medial frontal gyrus | 6 | CANTAB | Set shifting | ASTLICMD | -0.473 | 0.003 |
| Right superior medial frontal gyrus | 6 | CANTAB | Set shifting | ASTLM | -0.386 | 0.01 |
| Right supplementary motor area | 6 | CANTAB | Set Shifting | ASTLCMD | -0.323 | 0.048 |
| Right supplementary motor area | 6 | CANTAB | Set Shifting | ASTLDM | -0.363 | 0.025 |
| Right supplementary motor area | 6 | CANTAB | Set Shifting | ASTLICMD | -0.379 | 0.02 |
| Left middle frontal gyrus | 8 | NeuroTrax | Memory | Memory | 0.379 | 0.023 |
| Right middle frontal gyrus | 6 | NeuroTrax | Attention | Attention | 0.339 | 0.043 |
| Right superior medial frontal gyrus | 6 | Pen and Paper | RAVLT | Total | 0.393 | 0.016 |
| Right superior medial frontal gyrus | 6 | Pen and Paper | FAS | Semantic | 0.353 | 0.032 |
| Right supplementary motor area | 6 | Pen and Paper | RAVLT | Total | 0.394 | 0.016 |
| Right superior parietal gyrus | 7 | Pen and Paper | RAVLT | Total | 0.380 | 0.002 |

**Supplementary Table 5. Quality of life (SF-36 questionnaire) changes.**

| | *Control Group (N=33)* | | | *HBOT Group (N=29)* | | | ***Baseline Comparison P-value*** | ***Net Effect Size*** |
|---|---|---|---|---|---|---|---|---|
| | ***Baseline*** | ***Control*** | ***3 months P-value*** | ***Baseline*** | ***Post-HBOT*** | ***3 months P-value*** | | |
| Physical function | 86.97±15.41 | 89.25±12.99 | 0.55 | 84.28±14.16 | 90.71±8.70 | 0.08 | 0.53 | 0.66 |
| Physical limitations | 78.57±31.70 | 83.33±28.58 | 0.56 | 78.57±28.81 | 88.09±16.99 | 0.19 | 1 | 0.50 |
| Emotional limitations | 84.52±29.37 | 77.77±32.02 | 0.42 | 74.60±24.69 | 76.19±30.08 | 0.86 | 0.24 | 0.86 |
| Energy | 69.107±16.10 | 69.44±14.36 | 0.93 | 62.61±15.21 | 70.71±13.53 | 0.07 | 0.16 | 0.75 |
| Emotional well being | 77.14±16.10 | 76.74±14.71 | 0.92 | 71.80±14.88 | 78.28±13.53 | 0.14 | 0.24 | 0.71 |
| Social function | 87.05±17.17 | 88.42±16.60 | 0.76 | 84.52±20.50 | 86.30±20.11 | 0.77 | 0.64 | 0.69 |
| Pain | 81.42±19.92 | 83.61±19.34 | 0.68 | 79.16±17.12 | 85.83±13.65 | 0.17 | 0.68 | 0.65 |
| General health | 75.0±16.83 | 79.62±16.28 | 0.30 | 74.52±14.30 | 80.0±14.74 | 0.23 | 0.92 | 0.93 |

**Supplementary Table 6. Quality of life (SF-36 questionnaire) repeated measures analysis.**

| | Main Effect of Group | | Main Effect of Time | | Interaction Effect (Group_by_Time) | |
|---|---|---|---|---|---|---|
| | ***F*** | ***p-value*** | ***F*** | ***p-value*** | ***F*** | ***p-value*** |
| Physical function | 0.108 | 0.743 | 8.266 | 0.006 | 2.406 | 0.128 |
| Physical limitations | 0.096 | 0.758 | 2.025 | 0.162 | 0.580 | 0.450 |
| Emotional limitations | 0.379 | 0.541 | 0.296 | 0.589 | 0.814 | 0.372 |
| Energy | 0.274 | 0.604 | 6.281 | **0.016** | 6.908 | **0.012** |
| Emotional well being | 0.089 | 0.767 | 3.005 | 0.090 | 2.733 | 0.105 |
| Social function | 0.107 | 0.745 | 0.590 | 0.446 | 0.001 | 0.977 |
| Pain | 0.000 | 0.985 | 4.404 | **0.042** | 2.770 | 0.103 |
| General health | 0.010 | 0.920 | 7.690 | **0.008** | 0.087 | 0.769 |
